# Multidomain Cardiorenal-Metabolic Abnormalities Despite HbA1c <7% in Type 2 Diabetes: A Real-World Observational Study

**DOI:** 10.3390/diseases14090329

**Published:** 2026-09-09

**Authors:** Igna Andreea Diana, Petca Bianca-Lăcrimioara, Popovici Paula-Alexandra, Timea Claudia Ghitea, Roxana Daniela Brata, Daniela Florina Trifan, Mihaela Simona Popoviciu

**Affiliations:** 1 Doctoral School of Biological and Biomedical Sciences, University of Oradea, 1 University Street, 410087 Oradea, Romania; igna.andreeadiana@student.uoradea.ro (I.A.D.); criste.biancalacrimioara@student.uoradea.ro (P.B.-L.); cipleu.paulaalexandra@student.uoradea.ro (P.P.-A.); 2 Pharmacy Department, Faculty of Medicine and Pharmacy, University of Oradea, 1 University Street, 410087 Oradea, Romania; 3 Department of Medical Disciplines, Faculty of Medicine and Pharmacy, University of Oradea, 410087 Oradea, Romania; 4 Department of Clinical Discipline, Faculty of Medicine and Pharmacy, University of Oradea, 410068 Oradea, Romania; trifan.daniela17@yahoo.com; 5 Department of Preclinical Disciplines, Faculty of Medicine and Pharmacy, University of Oradea, 410028 Oradea, Romania; mihaela.popoviciu@didactic.uoradea.ro

**Keywords:** type 2 diabetes, HbA1c, multidomain abnormalities, LDL cholesterol, blood pressure, kidney function, obesity, real-world study

## Abstract

Background: Contemporary management of type 2 diabetes mellitus (T2DM) extends beyond glycemic control and requires comprehensive assessment of cardiovascular, renal, metabolic, and anthropometric factors. Nevertheless, clinical status is often summarized predominantly by glycated hemoglobin (HbA1c), potentially overlooking concurrent non-glycemic abnormalities. This study evaluated the prevalence and coexistence of cardiorenal-metabolic abnormalities among patients with HbA1c <7.0% at six months. Methods: This retrospective observational study included adults with T2DM routinely followed in a specialized outpatient diabetes clinic. The primary descriptive analysis included participants with HbA1c <7.0% at the six-month assessment (T1). Seven non-glycemic cardiorenal-metabolic domains were evaluated at T1: LDL cholesterol, HDL cholesterol, triglycerides, body mass index, blood pressure, estimated glomerular filtration rate, and urinary albumin-to-creatinine ratio. An unweighted descriptive count of domains outside study-defined analytical thresholds was calculated (range, 0–7). An exploratory Poisson regression model examined associations between selected baseline characteristics and the number of affected domains, excluding baseline variables that directly corresponded to components of the T1 count to minimize mathematical coupling. Results: Among 809 participants, 385 (47.6%) had HbA1c <7.0% at six months and were included in the primary descriptive analysis. Concurrent non-glycemic abnormalities were common, particularly for blood pressure, LDL cholesterol, triglycerides, body mass index, and HDL cholesterol. The median number of domains outside study-defined analytical thresholds was three (IQR 3–4), and only three participants (0.8%) had values within all seven thresholds. In the exploratory multivariable model, none of the evaluated demographic, glycemic, or treatment-related baseline characteristics was independently associated with the number of domains outside study-defined thresholds. Conclusions: HbA1c <7.0% at six months did not necessarily coincide with favorable values across other clinically relevant cardiovascular, metabolic, anthropometric, and kidney-related domains in patients with T2DM. The unweighted domain count provides a descriptive summary of concurrent non-glycemic abnormalities but should not be interpreted as a validated measure of risk, prognosis, or clinical risk stratification. These findings support comprehensive, individualized assessment beyond glycemic status alone.

## 1. Introduction

Type 2 diabetes mellitus (T2DM) is a complex multisystem disease associated with progressive metabolic dysfunction and a high burden of cardiovascular, kidney, and microvascular complications. Although glycated hemoglobin (HbA1c) remains an important marker of glycemic status, it does not fully characterize an individual’s broader cardiovascular, metabolic, and kidney profile. Patients with HbA1c values below conventional analytical cut-offs may still have obesity, dyslipidemia, hypertension, kidney involvement, or other clinically relevant risk factors [1,2,3].

Contemporary international guidelines have shifted from a predominantly glucose-centered approach toward integrated cardiovascular, kidney, metabolic, and weight management. Current recommendations emphasize individualized assessment and management of lipid levels, blood pressure, body weight, kidney involvement, and cardiovascular risk in addition to glycemic status [4,5,6,7,8].

Although HbA1c remains central to glycemic assessment, it does not replace evaluation of cardiovascular, metabolic, anthropometric, and kidney-related domains. Patients with HbA1c below a conventional analytical threshold may therefore still have concurrent non-glycemic abnormalities that warrant individualized assessment [9,10].

The literature and major clinical guidelines address cardiovascular, metabolic, anthropometric, and kidney-related measures as distinct components of T2DM care [4,5,6,11,12,13,14]. However, fewer real-world analyses have examined the simultaneous coexistence of multiple non-glycemic abnormalities among individuals selected using a conventional HbA1c cut-off. Describing these domains concurrently may provide information not captured by HbA1c alone while preserving the distinct clinical meaning of each measure.

The primary aim of the present study was to characterize the prevalence and coexistence of non-glycemic cardiorenal-metabolic abnormalities among adults with T2DM who had HbA1c <7.0% at the six-month assessment. Individual domains were analyzed separately, and an unweighted count of domains outside study-defined analytical thresholds was used only as a descriptive summary. A secondary, explicitly exploratory objective was to examine whether selected baseline characteristics were associated with the number of abnormalities observed at six months. The count was not intended as a prognostic score, risk-stratification instrument, or measure of overall disease control.

## 2. Materials and Methods

### 2.1. Study Design and Population

This retrospective observational study included 809 adults with type 2 diabetes mellitus (T2DM) who were evaluated at baseline (T0) and had planned clinical reassessment at approximately six months (T1). The full cohort was retained for the description of patient flow and for comparison of baseline characteristics according to six-month HbA1c status.

This retrospective study was conducted following approval by the Ethics Committee on 31 October 2025 and involved the secondary analysis of routinely collected clinical records covering the period from January 2016 to December 2021. Baseline (T0) was defined as the clinical and laboratory assessment performed at the index hospital admission, whereas T1 was defined as the corresponding follow-up assessment performed approximately six months after T0. All included participants had a T1 assessment performed after T0.

The study represents the retrospective component of a broader doctoral research project that also includes a prospective component. The prospective component and its associated data are not included in the present manuscript. The research database used for this analysis was accessed, constructed, and analyzed only after ethical approval had been obtained. No research-specific procedures, data extraction, database construction, or statistical analyses were performed before ethical approval.

The T0 and T1 assessments were conducted as part of routine clinical care and therefore preceded the ethical approval. These clinical observations were already available in the patients’ medical records before the present retrospective study commenced. Written informed consent for the secondary use of routinely collected clinical data was obtained in accordance with the Ethics Committee’s requirements and before participants’ data were entered into the research database. The relevant ethical approval and consent documentation can be made available confidentially to the Academic Editor upon request.

The primary descriptive analysis focused on participants with HbA1c <7.0% at T1 (n = 385), with the aim of characterizing the coexistence of non-glycemic cardiorenal-metabolic abnormalities among patients meeting this standardized glycemic threshold. Of the remaining 424 participants, 423 had HbA1c ≥7.0% at T1 and one participant had no available T1 HbA1c measurement.

Importantly, HbA1c <7.0% was used as a standardized analytical criterion and should not be interpreted as confirmation that an individualized glycemic target had been achieved. Contemporary diabetes management recommends individualized HbA1c goals according to age, diabetes duration, comorbidities, functional status, hypoglycemia risk, and life expectancy.

Because membership in the primary analytical subgroup was determined by HbA1c measured at follow-up, baseline characteristics of participants included in and excluded from the primary analysis were compared to characterize potential selection differences. Conditioning an analysis on a post-baseline variable can induce selection or collider bias when that variable is influenced by baseline characteristics or subsequent processes; this possibility was explicitly considered in the interpretation of the findings [15,16].

### 2.2. Eligibility Criteria

Eligibility for the source cohort required age ≥18 years, a confirmed diagnosis of T2DM, a routine baseline evaluation, and a planned clinical reassessment at approximately six months. HbA1c <7.0% at T1 was not a source-cohort eligibility criterion. Patients with type 1 diabetes mellitus, gestational diabetes, secondary forms of diabetes, active malignant disease, or an acute infectious or inflammatory condition during follow-up were excluded. Completeness requirements were analysis specific: the source cohort could retain limited missing data in variables not required for a particular analysis, whereas the primary descriptive analysis required a non-missing T1 HbA1c value <7.0% and non-missing T1 values for all seven domains (LDL-C, HDL-C, triglycerides, BMI, systolic and diastolic blood pressure, eGFR, and UACR). All 385 participants in the primary subgroup met these requirements. One member of the source cohort lacked T1 HbA1c and two lacked baseline UACR; available-case data were used for the corresponding baseline comparison.

### 2.3. Clinical and Laboratory Assessment

Clinical, anthropometric, biochemical, and therapeutic data were extracted retrospectively from electronic medical records on the database-access date reported in the study chronology. Demographic variables included age, sex, occupation, and diabetes duration. Body mass index (BMI) was calculated as weight in kilograms divided by height in meters squared (kg/m^2^).

Blood pressure values were obtained from routine office measurements recorded in the medical records at the corresponding study visit. Systolic and diastolic blood pressure were analyzed as documented. Because of the retrospective real-world design, information regarding the specific blood pressure device, cuff size, resting period, and number of repeated measurements was not consistently recorded and could not be retrospectively standardized. Accordingly, blood pressure values were treated as routine clinical measurements rather than protocol-standardized research measurements.

Laboratory variables included fasting plasma glucose, glycated hemoglobin (HbA1c), total cholesterol, LDL cholesterol, HDL cholesterol, triglycerides, serum creatinine, estimated glomerular filtration rate (eGFR), urinary albumin, and urinary creatinine. All measurements were obtained as part of routine clinical care and extracted from the laboratory reports available in the electronic medical records. HbA1c was expressed as %, glucose and lipid parameters as mg/dL, eGFR as mL/min/1.73 m^2^, and urinary albumin-to-creatinine ratio (UACR) as mg/mmol.

Renal function was evaluated using the eGFR value reported by the clinical laboratory together with serum creatinine. The eGFR values were not recalculated for the present study. Because the equation used for individual historical laboratory reports could not be consistently verified, the reported eGFR values were not attributed uniformly to a specific estimating equation. Current recommendations favor a race-free creatinine-based equation, but retrospectively applying a new equation without recalculating eGFR from the original creatinine, age, and sex data would introduce additional assumptions and was therefore avoided [17].

UACR was derived from urinary albumin and urinary creatinine measurements available in the clinical laboratory records and was expressed as mg/mmol. A value ≥3 mg/mmol (approximately ≥30 mg/g creatinine) was classified as elevated for the descriptive analysis. Because repeated confirmatory UACR measurements were not consistently available, an elevated UACR at T1 was interpreted as increased urinary albumin excretion at that assessment and not as confirmed persistent albuminuria.

Glucose-lowering medication classes were recorded independently and were not mutually exclusive; therefore, participants receiving combination therapy could contribute to more than one medication category. Detailed information on medication dose, treatment intensification, adherence, and changes in glucose-lowering, lipid-lowering, or antihypertensive therapy between T0 and T1 was not consistently available in the retrospective records. Accordingly, medication variables were analyzed as baseline treatment indicators and not as measures of treatment exposure during follow-up.

Detailed information regarding the analytical platform and assay methodology used for every historical laboratory determination was not consistently available in the retrospective records. Therefore, laboratory measurements were analyzed as routine clinical laboratory results and were not retrospectively assigned an analytical method that could not be verified from the source documentation.

### 2.4. Definition of Non-Glycemic Domains and Descriptive Domain Count

Seven non-glycemic cardiorenal-metabolic domains were evaluated at T1 using study-defined analytical thresholds selected for standardized descriptive comparison. The final set was not fixed before the current revision because the blood pressure threshold was updated from <140/90 to <130/80 mmHg in response to current guidance. The thresholds and the timing and basis of their selection are reported below and should not be interpreted as individualized therapeutic targets for every participant.

For LDL-C, contemporary diabetes guidelines recommend treatment intensity and goals according to ASCVD status and overall cardiovascular risk. In adults with diabetes aged 40–75 years at higher cardiovascular risk, an LDL-C goal of <70 mg/dL is recommended, whereas patients with established ASCVD generally require a more stringent goal. The LDL-C cut-off of 70 mg/dL was therefore used as a study-defined descriptive threshold, not as evidence that every participant had or had not achieved an individualized lipid goal.

Blood pressure goals in diabetes should be individualized according to cardiovascular and kidney risk, treatment tolerability, comorbidity burden, and patient characteristics. Current ADA guidance recommends an on-treatment goal of <130/80 mmHg when safely attainable and encourages consideration of a systolic goal <120 mmHg in people at high cardiovascular or kidney risk. The <130/80 mmHg cut-off used in the final analysis was selected during manuscript revision on this basis and differed from the cut-off used in the original analysis.

The HDL-C (<40 mg/dL in men and <50 mg/dL in women) and triglyceride (≥150 mg/dL) cut-offs were used as conventional cardiometabolic risk-marker thresholds rather than pharmacological treatment targets. BMI ≥30 kg/m^2^ was used to identify obesity according to the World Health Organization classification. An eGFR <60 mL/min/1.73 m^2^ and UACR ≥3 mg/mmol (approximately ≥30 mg/g) were used to identify kidney-related abnormalities at T1; these single-visit findings were not treated as therapeutic targets or, by themselves, as confirmation of chronic kidney disease [6,7,8,13,14].

UACR was assessed from the laboratory measurement available at T1 and expressed in mg/mmol. A UACR <3 mg/mmol was categorized as within the study-defined threshold, whereas UACR ≥3 mg/mmol was categorized as elevated. The cut-off corresponds approximately to 30 mg/g creatinine and identifies increased urinary albumin excretion. The domain was therefore termed elevated UACR rather than confirmed persistent albuminuria.

For the present analysis, one UACR measurement per participant at T1 was used. Repeated confirmatory measurements were not consistently available in the dataset. Consequently, an elevated UACR at T1 was not considered sufficient to establish persistent elevated UACR or chronic kidney disease on its own.

### 2.5. Study Outcomes

The primary outcome of the study was the prevalence and distribution of individual non-glycemic cardiorenal-metabolic domains outside study-defined analytical thresholds among participants with HbA1c <7.0% at the six-month assessment (T1). The concurrent presence of abnormalities across these domains was additionally summarized using an unweighted descriptive count ranging from 0 to 7. A secondary exploratory analysis examined the association between selected baseline demographic, glycemic, and treatment-related characteristics and the number of domains outside study-defined thresholds at T1. Baseline variables directly corresponding to components of the T1 domain count were excluded from this analysis to minimize mathematical coupling.

### 2.6. Statistical Analysis

Continuous variables were assessed for normality using the Shapiro–Wilk test and are presented as mean ± standard deviation or median with interquartile range, as appropriate. Baseline characteristics were compared between participants included in the primary analysis (HbA1c <7.0% at T1) and those excluded from it. Welch’s independent-samples t-test was used for approximately normally distributed continuous variables, the Mann–Whitney U test for skewed continuous variables, and the chi-square test with Yates continuity correction for binary categorical variables. These comparisons were intended to characterize potential selection differences rather than to identify causal determinants of six-month glycemic status. All tests were two-sided, and *p* < 0.05 was considered statistically significant.

The prevalence of each non-glycemic domain outside the study-defined analytical thresholds was summarized using descriptive statistics and graphically illustrated. The concurrent number of affected domains was calculated as an unweighted descriptive count ranging from 0 to 7. For the secondary exploratory analysis, a generalized linear model with a Poisson distribution and log link was used to examine associations between selected baseline characteristics and the number of domains outside study-defined thresholds at T1.

To minimize mathematical coupling, baseline variables directly corresponding to components of the T1 domain count (BMI, LDL-C, HDL-C, triglycerides, blood pressure/hypertension, eGFR, and UACR) were excluded from the multivariable model. The final model therefore included age, sex, diabetes duration, baseline HbA1c, and baseline glucose-lowering medication classes. Continuous predictors were standardized and expressed per one-standard-deviation increase. HC3 robust standard errors were used.

**Figure 1:**
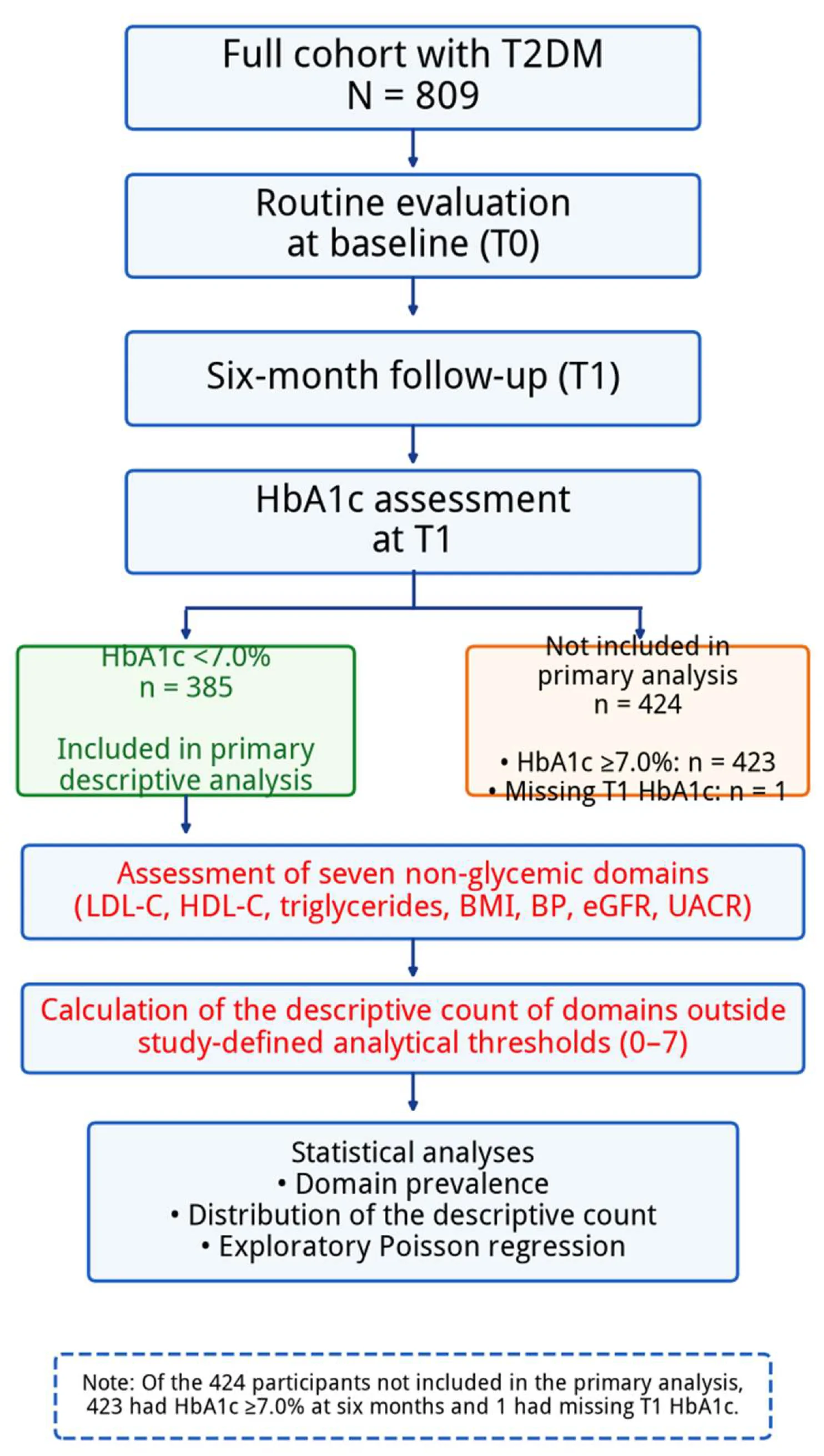
Study flow diagram and definition of the primary analytical cohort. HbA1c <7.0% at T1 was used as a standardized analytical criterion and does not necessarily indicate achievement of an individualized glycemic target.

Model dispersion was assessed using the deviance-to-degrees-of-freedom and Pearson chi-square-to-degrees-of-freedom ratios. Both ratios were substantially below 1 (deviance/df = 0.510; Pearson χ^2^/df = 0.458), indicating marked underdispersion relative to the Poisson variance assumption. Negative binomial regression was not used because it addresses overdispersion rather than underdispersion. The Poisson model was retained only as a secondary exploratory analysis of the conditional mean, with HC3 robust standard errors and cautious interpretation; it was not used for prediction, variable selection, or causal inference. Results are reported as adjusted incidence rate ratios (IRRs) with 95% confidence intervals. All analyses were performed using IBM SPSS Statistics version 30 (IBM Corp., Armonk, NY, USA); tests were two-sided and *p* < 0.05 was considered statistically significant. Figure 1 presents the study flow diagram.

## 3. Results

### 3.1. Patient Flow and Baseline Characteristics According to Inclusion in the Primary Analysis

The full cohort comprised 809 participants with T2DM. At the six-month assessment, 385 participants had HbA1c <7.0% and were included in the primary descriptive analysis of non-glycemic cardiorenal-metabolic abnormalities. Among the 424 participants not included in this analysis, 423 had HbA1c ≥7.0%, while one participant had no available T1 HbA1c measurement (Figure 1).

Baseline characteristics of the full cohort and according to subsequent inclusion in the primary analysis are presented in Table 1. Participants with HbA1c <7.0% at T1 were slightly older than those excluded from the primary analysis (64.04 ± 9.83 vs. 62.65 ± 10.19 years; *p* = 0.047). As expected, they had substantially lower baseline HbA1c [7.00% (IQR 6.50–7.40) vs. 8.50% (IQR 7.70–9.60); *p* < 0.001].

Baseline insulin treatment was also less frequent among participants subsequently included in the HbA1c <7.0% subgroup (17.7% vs. 33.0%; *p* < 0.001). SGLT2 inhibitor use showed a borderline between-group difference (31.4% vs. 38.2%; *p* = 0.052), whereas no statistically significant differences were observed for sex, diabetes duration, BMI, LDL-C, HDL-C, triglycerides, eGFR, UACR, hypertension, biguanide use, sulfonylurea use, DPP-4 inhibitor use, or GLP-1 receptor agonist use.

These findings indicate that selection according to six-month HbA1c status was associated particularly with baseline glycemic severity and insulin treatment, while most baseline non-glycemic metabolic and renal characteristics were broadly comparable between groups.

### 3.2. Distribution of Non-Glycemic Domains Outside Study-Defined Analytical Thresholds

Among participants selected because HbA1c was <7.0% at six months, most had one or more clinically relevant non-glycemic cardiorenal-metabolic abnormalities.

Blood pressure was the most frequently affected domain, with 332 participants (86.2%) having values outside the study-defined analytical threshold. LDL cholesterol was outside the study-defined threshold in 254 participants (66.0%), while triglycerides were elevated in 167 participants (43.4%).

Kidney-related abnormalities were less frequent than blood pressure or lipid abnormalities but remained common. Elevated UACR was observed in 122 participants (31.7%), whereas 78 participants (20.3%) had eGFR <60 mL/min/1.73 m^2^.

**Figure 2:**
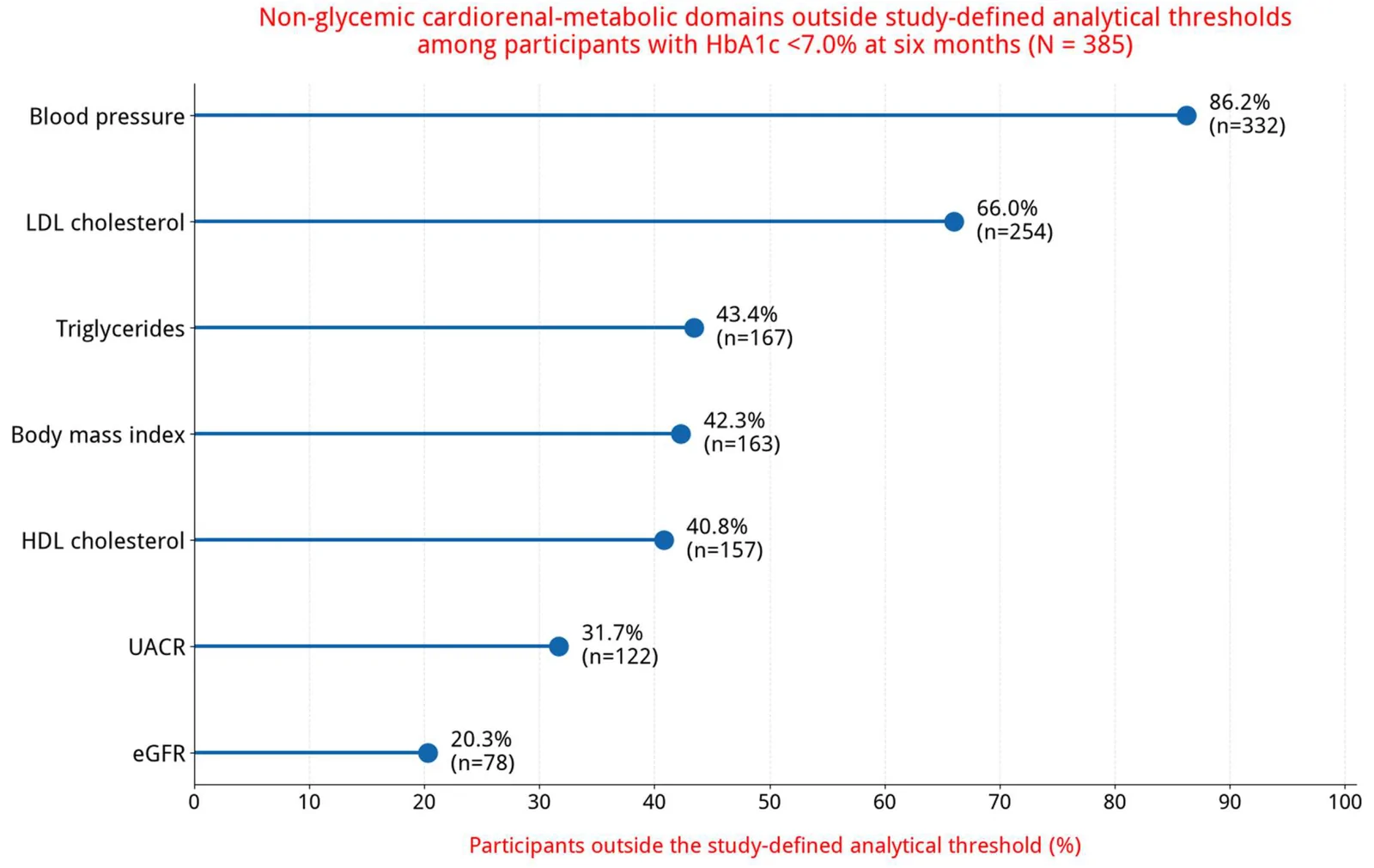
Prevalence of non-glycemic cardiorenal-metabolic domains outside study-defined analytical thresholds among participants with HbA1c <7.0% at the six-month assessment (N = 385). Blood pressure was the most frequently affected domain (86.2%), followed by LDL-C (66.0%), triglycerides (43.4%), BMI (42.3%), HDL-C (40.8%), elevated UACR (31.7%), and reduced eGFR (20.3%). The thresholds were applied for descriptive purposes and should not be interpreted as individualized therapeutic targets.

These results show that HbA1c <7.0% frequently coexisted with lipid, blood pressure, anthropometric, and kidney-related abnormalities. The prevalence of each domain outside its study-defined threshold is presented in Table 2 and Figure 2.

### 3.3. Coexistence of Multiple Non-Glycemic Abnormalities

Complete multidomain alignment with all seven study-defined analytical thresholds was uncommon. Only 3 participants (0.8%) had no domains outside the study-defined thresholds, whereas 28 (7.3%) had one affected domain. Most participants had multiple concurrent abnormalities: 354 (91.9%) had at least two domains outside the study-defined thresholds, 295 (76.6%) had at least three, and 168 (43.6%) had four or more.

**Figure 3:**
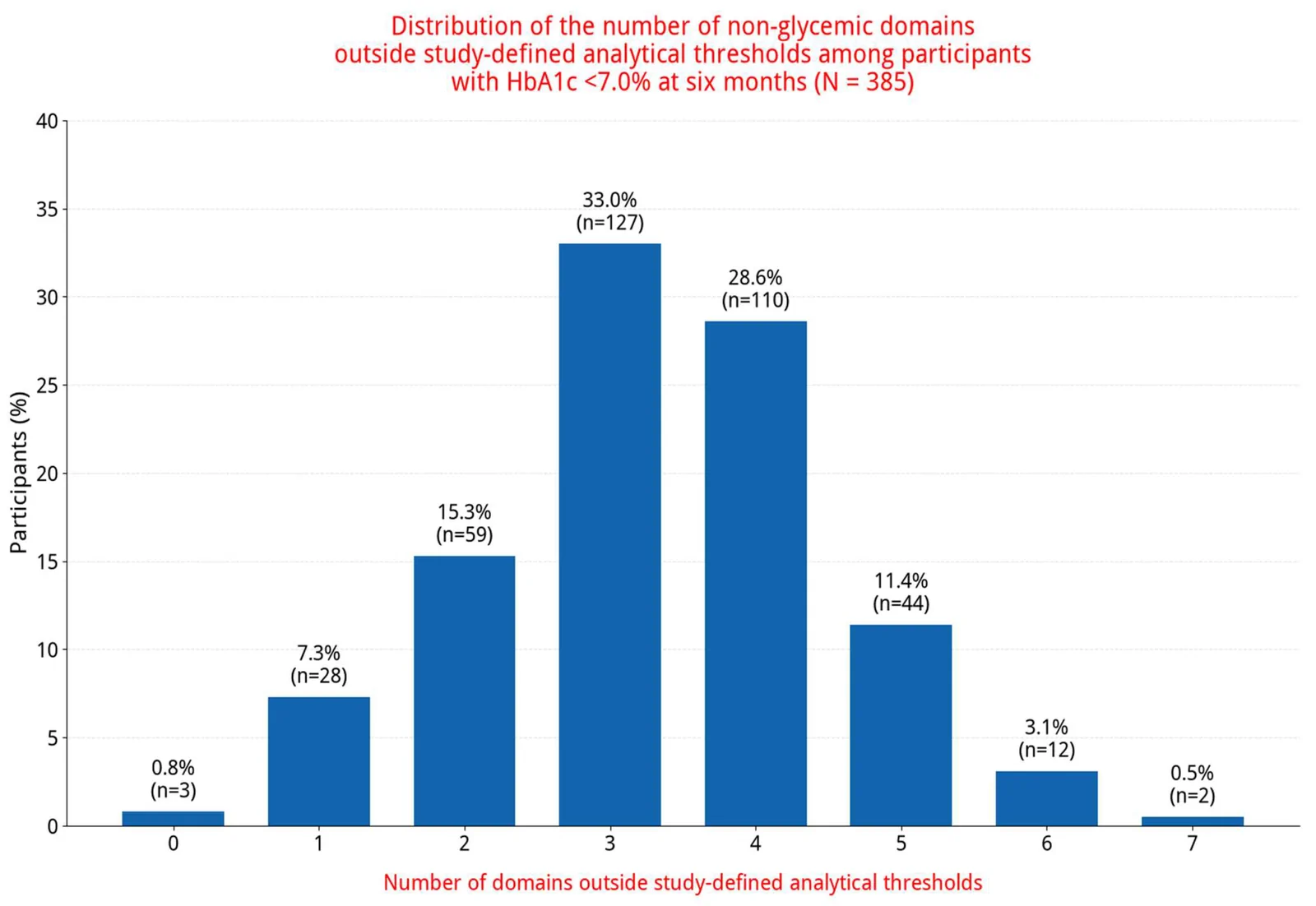
Distribution of the descriptive count of non-glycemic domains outside study-defined analytical thresholds among participants with HbA1c <7.0% at the six-month assessment (T1). The figure shows the frequency distribution of the unweighted number of concurrently assessed domains outside study-defined thresholds, ranging from 0 to 7. The count is presented for descriptive purposes only and should not be interpreted as a validated risk score or prognostic instrument.

The mean number of domains outside study-defined analytical thresholds was 3.31 ± 1.24, with a median of 3 (IQR 3–4). The most frequent count was 3 domains, observed in 127 participants (33.0%), followed by 4 domains in 110 participants (28.6%). Fourteen participants (3.6%) had six or seven domains outside the study-defined analytical thresholds. The complete distribution is presented in Table 3 and Figure 3.

The distribution of the number of non-glycemic domains outside study-defined analytical thresholds among participants with HbA1c <7.0% at the six-month assessment is presented in Figure 3.

### 3.4. Secondary Exploratory Analysis of Baseline Factors Associated with the Number of Domains Outside Study-Defined Analytical Thresholds

To minimize mathematical coupling, baseline variables corresponding directly to components of the T1 descriptive domain count (BMI, LDL-C, HDL-C, triglycerides, blood pressure/hypertension, eGFR, and UACR) were excluded from the primary exploratory multivariable model. The model therefore included age, sex, diabetes duration, baseline HbA1c, and baseline glucose-lowering medication classes.

None of the evaluated baseline characteristics were independently associated with the number of domains outside study-defined thresholds at T1 (all *p* > 0.05). Male sex (adjusted IRR 0.933, 95% CI 0.866–1.004; *p* = 0.064) and SGLT2 inhibitor use (adjusted IRR 0.930, 95% CI 0.858–1.008; *p* = 0.078) also did not meet the defined statistical significance criterion; these *p* values were not interpreted as evidence of trends.

The dispersion ratios indicated marked underdispersion (deviance/df = 0.510; Pearson χ^2^/df = 0.458). Accordingly, the exploratory model should be interpreted cautiously. After avoiding direct overlap between baseline predictors and components of the outcome, the available demographic, glycemic, and baseline treatment indicators did not independently explain the number of concurrent abnormalities at six months (Table 4).

## 4. Discussion

### 4.1. Principal Findings

The present study shows that HbA1c <7.0% at six months did not necessarily coincide with favorable values across other clinically relevant cardiorenal-metabolic domains in patients with T2DM. Among participants meeting this standardized glycemic criterion, abnormalities across lipid, anthropometric, blood pressure, and kidney-related domains remained highly prevalent. Only a small minority had values within all seven study-defined analytical thresholds.

To characterize this discrepancy, we calculated an unweighted descriptive count of non-glycemic domains outside study-defined analytical thresholds. The distribution of this count showed that most participants had multiple concurrent cardiorenal-metabolic abnormalities despite having HbA1c <7.0% at six months. These findings illustrate that HbA1c alone does not capture the coexistence of abnormalities across lipid, anthropometric, blood pressure, and renal domains in routine clinical practice.

An important consideration is that the primary analytical subgroup was defined according to HbA1c measured at the six-month assessment. Comparison with participants excluded from this subgroup demonstrated that those with HbA1c <7.0% at T1 had lower baseline HbA1c and were less frequently treated with insulin at baseline. These differences suggest that the analyzed subgroup represented a clinically selected phenotype with more favorable baseline glycemic status and/or lower treatment complexity. Conversely, most baseline non-glycemic metabolic and renal measures, including BMI, lipid parameters, eGFR, UACR, and hypertension prevalence, did not differ significantly between groups. These findings improve characterization of the selection process but do not eliminate the possibility of selection bias [15,16].

These findings reinforce the concept that type 2 diabetes should no longer be viewed as a glucose-centered disorder but rather as a multifactorial cardiometabolic disease requiring simultaneous optimization of several interconnected biological systems.

### 4.2. Concurrent Abnormalities Beyond Glycemic Status

Contemporary management of type 2 diabetes includes cardiovascular risk reduction, kidney protection, weight management, and metabolic assessment in addition to glycemic management. Current ADA, ESC, ADA–KDIGO, and KDIGO guidance supports individualized, multifactorial care [4,5,6,7,8].

In this cohort, more than four-fifths of participants had blood pressure values outside the study-defined analytical threshold, two-thirds had LDL-C ≥70 mg/dL, and approximately two-fifths had elevated triglycerides or obesity. Kidney-related abnormalities were also present in a clinically relevant proportion of participants.

These observations show that HbA1c status and the profile of other cardiometabolic and kidney-related measures are distinct dimensions. Reliance on HbA1c alone may therefore underrecognize concurrent abnormalities that warrant individualized clinical assessment [4,5,6].

### 4.3. Clinical Interpretation of Concurrent Cardiorenal-Metabolic Abnormalities

The findings should be interpreted at the level of the individual clinical domains, not as support for a new composite clinical instrument. The descriptive count summarizes coexistence among participants with HbA1c <7.0% but does not assign differential clinical or prognostic weight to its components.

The evaluated domains are clinically heterogeneous. LDL-C and blood pressure are modifiable cardiovascular risk factors with guideline-based goals; BMI reflects anthropometric status; HDL-C and triglycerides are cardiometabolic risk markers; and eGFR and UACR are used in kidney disease detection, classification, and risk assessment [4,6,7,8,13,14,18,19].

The high frequency of concurrent abnormalities observed in this cohort nevertheless emphasizes the importance of comprehensive assessment beyond HbA1c. Among participants with HbA1c <7.0% at six months, abnormalities in blood pressure, lipid parameters, adiposity, and kidney-related measures frequently coexisted. These findings support evaluation of each domain according to the patient’s individual cardiovascular and kidney risk profile and the corresponding guideline recommendations, rather than management based on the numerical domain count itself [4,5,6,7,8,20,21,22,23].

Reduced eGFR and elevated UACR indicate kidney involvement and are used for CKD detection, classification, and risk assessment rather than as therapeutic targets. Their equal numerical contribution to the descriptive count does not imply equivalent clinical importance or prognostic significance.

### 4.4. Comparison with Previous Literature

Randomized trials, observational studies, and contemporary guidelines emphasize that management of T2DM should extend beyond glycemic status to cardiovascular and kidney risk reduction. The Steno-2 program provided early evidence supporting intensive multifactorial intervention, and current ADA, ESC, ADA–KDIGO, and KDIGO guidance reinforces individualized management of cardiovascular and kidney risk [4,5,6,7,8,20,24,25,26,27,28,29].

Our findings are consistent with this broader clinical framework by showing that HbA1c <7.0% at six months frequently coexisted with abnormalities across other clinically relevant domains. In particular, blood pressure and LDL-C were commonly outside the study-defined analytical thresholds, while abnormalities in triglycerides, BMI, HDL-C, UACR, and eGFR were also observed. The frequent coexistence of several abnormalities within the same individuals further illustrates that glycemic status alone provides an incomplete description of the broader cardiometabolic and kidney profile [20,21,22,23,30].

Unlike studies evaluating the prognostic effects of individual risk factors or validated cardiovascular and kidney risk models, the present study was not designed to estimate risk or predict clinical outcomes. The unweighted domain count was used solely as a descriptive summary of concurrent abnormalities and does not imply equivalent clinical importance of its individual components. Accordingly, our findings should be viewed as real-world descriptive evidence supporting comprehensive, individualized assessment of cardiovascular, metabolic, and kidney-related domains in patients with type 2 diabetes, rather than as validation of a new composite risk-stratification instrument.

### 4.5. Strengths and Limitations

The principal strength of this study is the simultaneous evaluation of several routinely available non-glycemic cardiorenal-metabolic domains in a real-world cohort of patients with T2DM and HbA1c <7.0% at six months. The relatively large initial cohort and the availability of demographic, metabolic, renal, and treatment-related variables allowed characterization of the coexistence of abnormalities that may not be apparent when glycemic status is considered in isolation. In addition, comparison of the primary analytical cohort with participants who had HbA1c ≥7.0% at T1 improved transparency regarding cohort selection and potential selection effects.

Several limitations should be acknowledged. First, the retrospective, single-center observational design precludes causal inference and may limit the generalizability of the findings. The unweighted domain count was constructed solely as a descriptive summary of concurrent abnormalities and has not been validated as a composite clinical, prognostic, or risk-stratification instrument. Its components are clinically heterogeneous and should not be assumed to have equivalent therapeutic or prognostic importance. In particular, LDL-C and blood pressure represent modifiable risk factors with guideline-based treatment goals, BMI reflects anthropometric status, HDL-C primarily represents a cardiometabolic risk marker, and reduced eGFR and elevated UACR indicate kidney involvement rather than therapeutic targets themselves.

Second, the thresholds used in the descriptive analysis were standardized analytical cut-offs rather than fully individualized therapeutic targets. In clinical practice, LDL-C, blood pressure, and glycemic goals should be individualized according to ASCVD status, cardiovascular and kidney risk, age, comorbidities, frailty, treatment tolerability, risk of hypoglycemia, and other patient-specific characteristics. Accordingly, classification of a value as outside a study-defined analytical threshold in this study should not be interpreted as evidence that an individual patient failed to achieve his or her clinically appropriate treatment goal.

Third, UACR was classified using the measurement available at T1. Because repeated confirmatory measurements were not consistently available, UACR ≥3 mg/mmol was interpreted as elevated urinary albumin excretion at that assessment and not as confirmed persistent albuminuria. Similarly, because data were obtained retrospectively from routine clinical records, detailed information regarding standardized blood pressure measurement procedures, laboratory analytical platforms, and the eGFR equation used for all historical measurements could not be consistently verified. These factors may have introduced measurement heterogeneity and affected threshold-based classification.

Fourth, an important methodological consideration is the potential for mathematical coupling between repeated measurements of the same clinical domains. Baseline BMI, LDL-C, HDL-C, triglycerides, blood pressure/hypertension, eGFR, and UACR directly correspond to components of the descriptive domain count assessed at T1. Including these variables as predictors could therefore generate associations reflecting within-person tracking of the same measures over time rather than independent determinants of the T1 count. To minimize this problem, these variables were excluded from the primary exploratory multivariable model.

Fifth, the exploratory Poisson regression had limited explanatory value. None of the included variables was independently associated with the domain count, medication categories were non-mutually exclusive, and treatment exposure, intensification, dose changes, adherence, lipid-lowering therapy, and antihypertensive modifications during follow-up were unavailable. In addition, the count showed marked underdispersion relative to the Poisson assumption. The model was retained only to report whether a small set of readily available baseline characteristics explained variation in the count after removal of mathematically coupled predictors. Its findings should be regarded as secondary and hypothesis-generating, not as evidence of treatment effects, comparative efficacy, prediction, or causal association.

Finally, the primary analytical cohort was defined by HbA1c <7.0% at the six-month assessment, a post-baseline characteristic that may itself have been influenced by baseline disease severity, treatment selection and intensification, adherence, and other measured or unmeasured factors. Conditioning the primary analysis on this follow-up variable may therefore introduce selection or collider bias [15,16]. To improve transparency, baseline characteristics of the 385 participants included in the primary analysis were compared with those of the 424 participants not included because of HbA1c ≥7.0% or missing T1 HbA1c data. Differences were particularly evident for baseline HbA1c and insulin treatment. The findings should therefore be interpreted as descriptive of the subgroup with HbA1c <7.0% at six months and should not be generalized to the entire T2DM cohort or interpreted as determinants of glycemic target achievement. Prospective multicenter studies incorporating individualized treatment targets, longitudinal treatment changes, adherence, and cardiovascular, kidney, and mortality outcomes are required to establish the clinical significance of the observed patterns of concurrent abnormalities.

## 5. Conclusions

Among patients with type 2 diabetes and HbA1c <7.0% at six months, abnormalities across multiple non-glycemic cardiorenal-metabolic domains remained common, particularly for blood pressure, LDL cholesterol, triglycerides, BMI, and HDL cholesterol. These findings demonstrate that HbA1c <7.0% does not necessarily coincide with favorable values across other clinically relevant cardiovascular, metabolic, and kidney-related domains.

The unweighted domain count provides a descriptive summary of concurrent abnormalities and should not be interpreted as a validated measure of risk, overall disease control, prognosis, or clinical risk stratification. Individual domains remain clinically distinct and should be assessed according to patient-specific cardiovascular and kidney risk and individualized treatment goals.

These real-world findings support comprehensive assessment beyond glycemic status alone. Prospective multicenter studies incorporating individualized therapeutic targets, treatment changes and adherence, and cardiovascular, kidney, and mortality outcomes are needed to determine the clinical significance and prognostic implications of different patterns of concurrent cardiorenal-metabolic abnormalities.

## Figures and Tables

**Table 1:** Baseline characteristics of the full cohort according to six-month HbA1c status.

Variable	Full Cohort (N = 809)	Included in Primary Analysis: HbA1c <7.0% (n = 385)	Excluded from Primary Analysis (n = 424) *	*p* Value
Age, years	63.31 ± 10.04	64.04 ± 9.83	62.65 ± 10.19	0.047
Female sex, n (%)	379 (46.8%)	172 (44.7%)	207 (48.8%)	0.267
Diabetes duration, years	6.00 (4.00–10.00)	6.00 (3.00–10.00)	7.00 (4.00–11.00)	0.163
BMI, kg/m^2^	30.41 ± 5.44	30.13 ± 5.10	30.67 ± 5.72	0.151
Baseline HbA1c, %	7.70 (6.90–8.80)	7.00 (6.50–7.40)	8.50 (7.70–9.60)	<0.001
LDL-C, mg/dL	98.14 ± 33.17	98.92 ± 32.18	97.43 ± 34.06	0.522
HDL-C, mg/dL	44.99 ± 12.43	45.43 ± 12.53	44.58 ± 12.34	0.331
Triglycerides, mg/dL	149.00 (104.00–204.00)	150.00 (106.00–198.00)	146.00 (100.75–208.00)	0.871
eGFR, mL/min/1.73 m^2^	79.12 ± 19.51	78.85 ± 19.48	79.36 ± 19.55	0.709
UACR †, mg/mmol	2.20 (1.10–8.64)	2.20 (1.10–9.39)	2.20 (1.10–8.06)	0.518
Hypertension, n (%)	450 (55.6%)	221 (57.4%)	229 (54.0%)	0.368
Glucose-lowering medication				
Insulin, n (%)	208 (25.7%)	68 (17.7%)	140 (33.0%)	<0.001
Biguanides, n (%)	474 (58.6%)	235 (61.0%)	239 (56.4%)	0.202
Sulfonylureas, n (%)	71 (8.8%)	29 (7.5%)	42 (9.9%)	0.286
DPP-4 inhibitors, n (%)	138 (17.1%)	60 (15.6%)	78 (18.4%)	0.333
GLP-1 receptor agonists, n (%)	132 (16.3%)	71 (18.4%)	61 (14.4%)	0.143
SGLT2 inhibitors, n (%)	283 (35.0%)	121 (31.4%)	162 (38.2%)	0.052

Of the 424 participants excluded from the primary analysis, 423 had HbA1c ≥7.0% at T1 and one had missing T1 HbA1c. † Baseline UACR was unavailable for two participants; the UACR comparison used available cases. *p* values compare participants included versus excluded from the primary analysis and were calculated using Welch’s independent-samples t-test, the Mann–Whitney U test, or the chi-square test with Yates continuity correction, as appropriate. * The excluded group comprised 423 participants with HbA1c ≥7.0% and one participant with a missing follow-up HbA1c value.

**Table 2:** Study-defined analytical thresholds used to describe individual cardiorenal-metabolic domains (N = 385).

Domain	Study-Defined Analytical Threshold	Interpretation	Within Threshold, n (%)	Outside Threshold, n (%)
LDL-C	<70 mg/dL	Study-defined analytical threshold; individualized LDL-C goals depend on ASCVD risk/status	131 (34.0)	254 (66.0)
HDL-C	≥40 mg/dL in men; ≥50 mg/dL in women	Cardiometabolic risk marker; not a direct pharmacological treatment target	228 (59.2)	157 (40.8)
Triglycerides	<150 mg/dL	Conventional cardiometabolic risk-marker threshold; not an individualized treatment target	218 (56.6)	167 (43.4)
BMI	<30 kg/m^2^	Absence of obesity according to BMI classification; not an individualized treatment target	222 (57.7)	163 (42.3)
Blood pressure	<130/80 mmHg *	Study-defined on-treatment cut-off selected during revision; individualized according to cardiovascular and kidney risk	53 (13.8)	332 (86.2)
eGFR	≥60 mL/min/1.73 m^2^	Marker of kidney function; not a therapeutic target	307 (79.7)	78 (20.3)
UACR	<3 mg/mmol (~30 mg/g)	Study-defined threshold for urinary albumin excretion; UACR ≥3 mg/mmol categorized as elevated	263 (68.3)	122 (31.7)

All domains were assessed at the six-month evaluation (T1). Percentages were calculated using the primary analytical cohort (N = 385) as the denominator. The thresholds were applied for descriptive purposes and should not be interpreted as individualized therapeutic targets. Blood pressure goals should be individualized according to cardiovascular and kidney risk, comorbidities, treatment tolerability, and patient characteristics. UACR was assessed using the available T1 measurement and expressed in mg/mmol. Because repeated confirmatory measurements were not consistently available, UACR ≥3 mg/mmol was described as elevated UACR rather than confirmed persistent albuminuria. ASCVD, atherosclerotic cardiovascular disease; BMI, body mass index; eGFR, estimated glomerular filtration rate; HDL-C, high-density lipoprotein cholesterol; LDL-C, low-density lipoprotein cholesterol; UACR, urinary albumin-to-creatinine ratio. * Blood pressure was classified as within the study-defined threshold only when both systolic blood pressure was <130 mmHg and diastolic blood pressure was <80 mmHg.

**Table 3:** Distribution of the number of non-glycemic domains outside study-defined analytical thresholds among participants with HbA1c <7.0% at six months (N = 385).

Number of Domains Outside Study-Defined Thresholds	Patients, n	Percentage, %
0	3	0.8
1	28	7.3
2	59	15.3
3	127	33.0
4	110	28.6
5	44	11.4
6	12	3.1
7	2	0.5
Total	385	100.0

The mean number of non-glycemic domains outside study-defined analytical thresholds was 3.31 ± 1.24, with a median of 3 (IQR 3–4). Only 3 participants (0.8%) had no domains outside the study-defined thresholds, whereas 354 (91.9%) had at least two, 295 (76.6%) had at least three, and 168 (43.6%) had four or more concurrent abnormalities.

**Table 4:** Exploratory multivariable Poisson regression of baseline factors associated with the number of non-glycemic domains outside study-defined analytical thresholds at six months (N = 385).

Baseline Factor	Adjusted IRR	95% CI	*p* Value
Age, per 1 SD increase	1.017	0.977–1.059	0.407
Male sex	0.933	0.866–1.004	0.064
Diabetes duration, per 1 SD increase	1.025	0.986–1.065	0.215
Baseline HbA1c, per 1 SD increase	1.016	0.976–1.057	0.432
Insulin	0.949	0.857–1.050	0.311
Biguanides	1.007	0.933–1.087	0.861
Sulfonylureas	0.961	0.847–1.089	0.532
DPP-4 inhibitors	1.032	0.923–1.154	0.579
GLP-1 receptor agonists	1.048	0.963–1.139	0.278
SGLT2 inhibitors	0.930	0.858–1.008	0.078

IRR, incidence rate ratio; CI, confidence interval; DPP-4, dipeptidyl peptidase-4; GLP-1, glucagon-like peptide-1; SGLT2, sodium-glucose cotransporter-2. Age, diabetes duration, and baseline HbA1c were standardized and are reported per one-standard-deviation increase. Baseline variables corresponding directly to components of the T1 descriptive domain count (BMI, LDL-C, HDL-C, triglycerides, blood pressure/hypertension, eGFR, and UACR) were excluded to minimize mathematical coupling. The model included 385 complete cases and used HC3 robust standard errors. Dispersion ratios below 1 (deviance/df = 0.510; Pearson χ^2^/df = 0.458) indicated marked underdispersion; therefore, the results are exploratory and should be interpreted cautiously.

## Data Availability

The data supporting the findings of this study are available from the corresponding authors upon reasonable request and subject to applicable ethical and privacy restrictions.

## Abbreviations

The following abbreviations are used in this manuscript:

Abbreviation	Definition
ASCVD	Atherosclerotic cardiovascular disease
BMI	Body mass index
BP	Blood pressure
CI	Confidence interval
CKD	Chronic kidney disease
DPP-4	Dipeptidyl peptidase-4
eGFR	Estimated glomerular filtration rate
GLP-1	Glucagon-like peptide-1
HbA1c	Glycated hemoglobin
HDL-C	High-density lipoprotein cholesterol
IQR	Interquartile range
IRR	Incidence rate ratio
LDL-C	Low-density lipoprotein cholesterol
SD	Standard deviation
SGLT2	Sodium–glucose cotransporter-2
T0	Baseline assessment
T1	Six-month follow-up assessment
T2DM	Type 2 diabetes mellitus
UACR	Urinary albumin-to-creatinine ratio
